# Supplementary material for: Recurrent Implantation Failure: Bioinformatic Discovery of Biomarkers and Identification of Metabolic Subtypes
Source: Int J Mol Sci. 2023 Aug 30;24(17):13488. doi: 10.3390/ijms241713488 (PMC10487894; doi:10.3390/ijms241713488)
Supplement: Supplementary file 1 [file ijms-24-13488-s001.zip › ijms-2531228-supplementary.pdf]

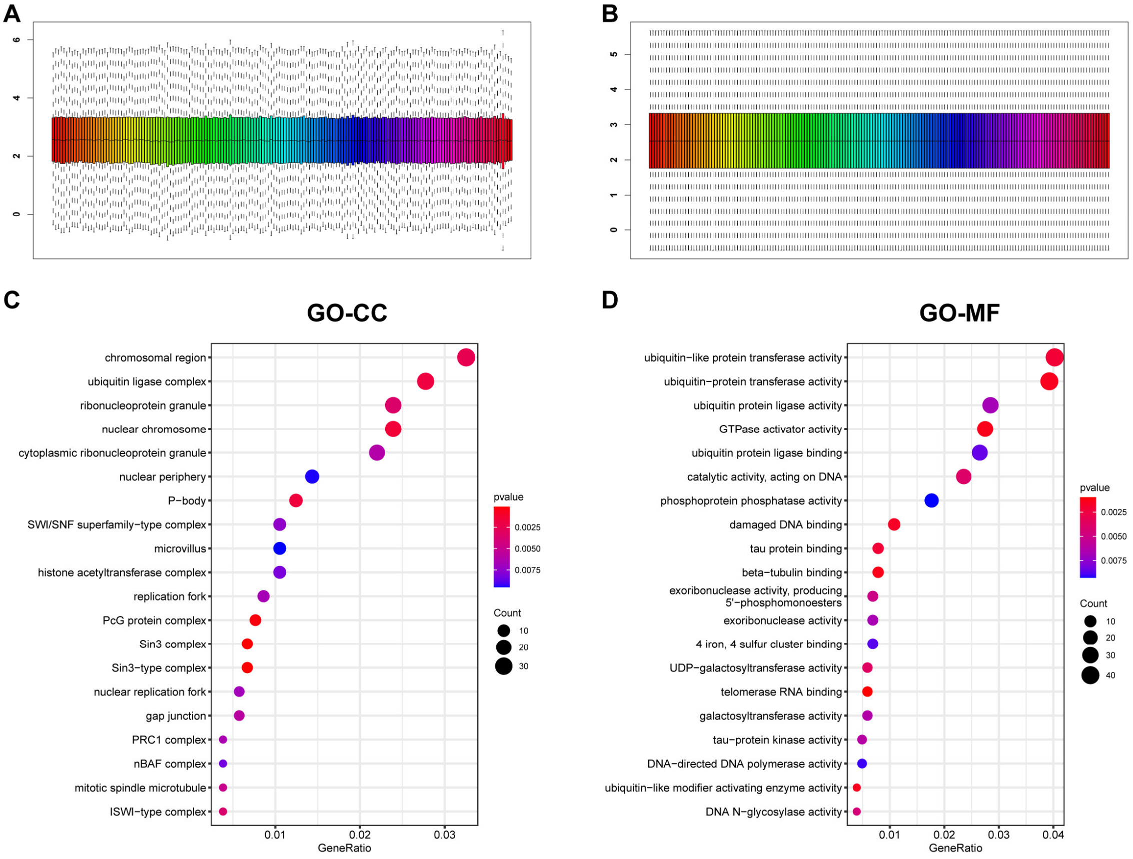

Figure S1. Data processing and analyses of functional enrichment of RIF-DEGs. Gene expression level statistics of the dataset before (A) and after (B) de-batching. The colors represent samples from three different datasets, respectively. (C-D) Main CCs, and MFs enriched by RIF-related DEGs. Each term's P value is colored according to the legend. Abbreviations: CC: cellular component, MF: molecular function, DEG: differentially expressed genes, RIF: recurrent implantation failure.

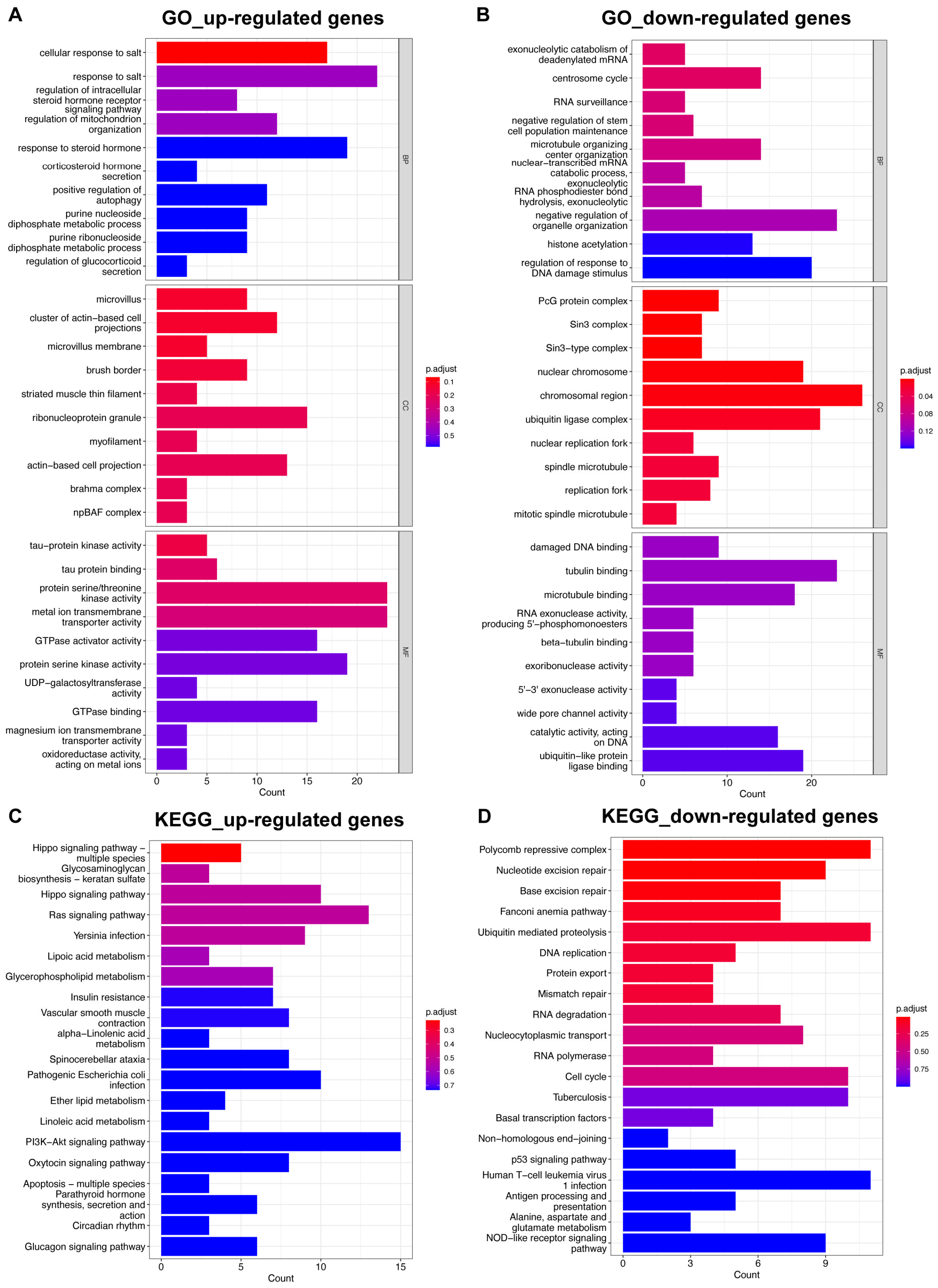

Figure S2. Functional enrichment analyses of separated upregulated and downregulated RIF-DEGs. GO enrichment analyses of (A) upregulated and (B) downregulated RIF-related DEGs. KEGG pathways enriched by (C) upregulated and (D) downregulated RIF-related DEGs. Each term's P value is colored according to the legend. Abbreviations: BP: biological process, CC: cellular component, DEG: differentially expressed genes, GO: Gene Ontology, MF: molecular function, KEGG: Kyoto Encyclopedia of Genes and Genomes, RIF: recurrent implantation failure.

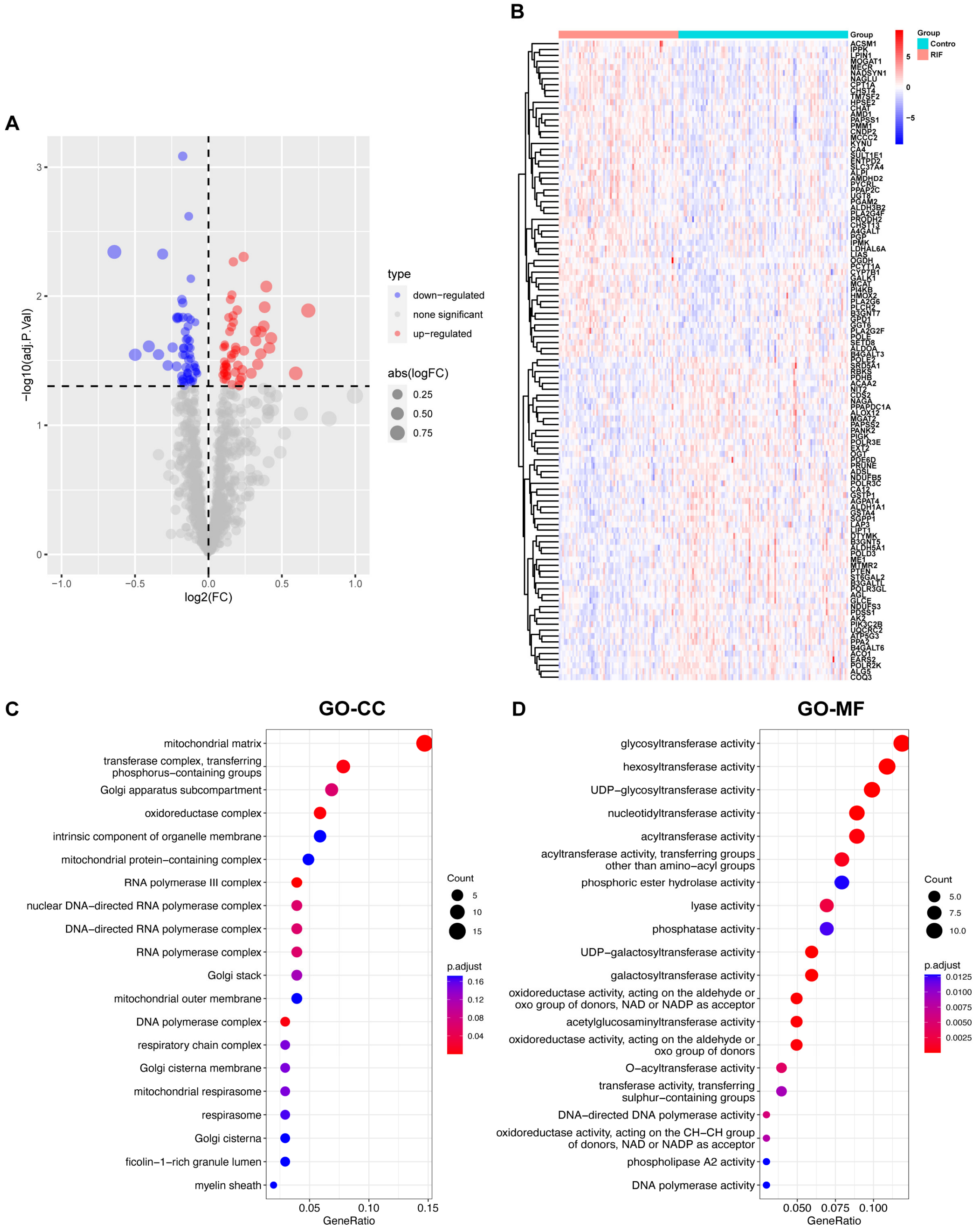

Figure S3. Metabolism-related DEGs and enriched items in CC and MF. (A) Metabolism-related DEGs volcano plot with log2FoldChange in the horizontal coordinate and -log10(adjust P value) in the vertical coordinate. (B) Heatmap of metabolism-related DEG expression levels: red indicates high gene expression, and blue indicates low gene expression. Enriched items in (C) CC and (D) MF analysis. Each term's P value is colored according to the legend. Abbreviations: CC: cellular component, MF: molecular function, DEG: differentially expressed genes.

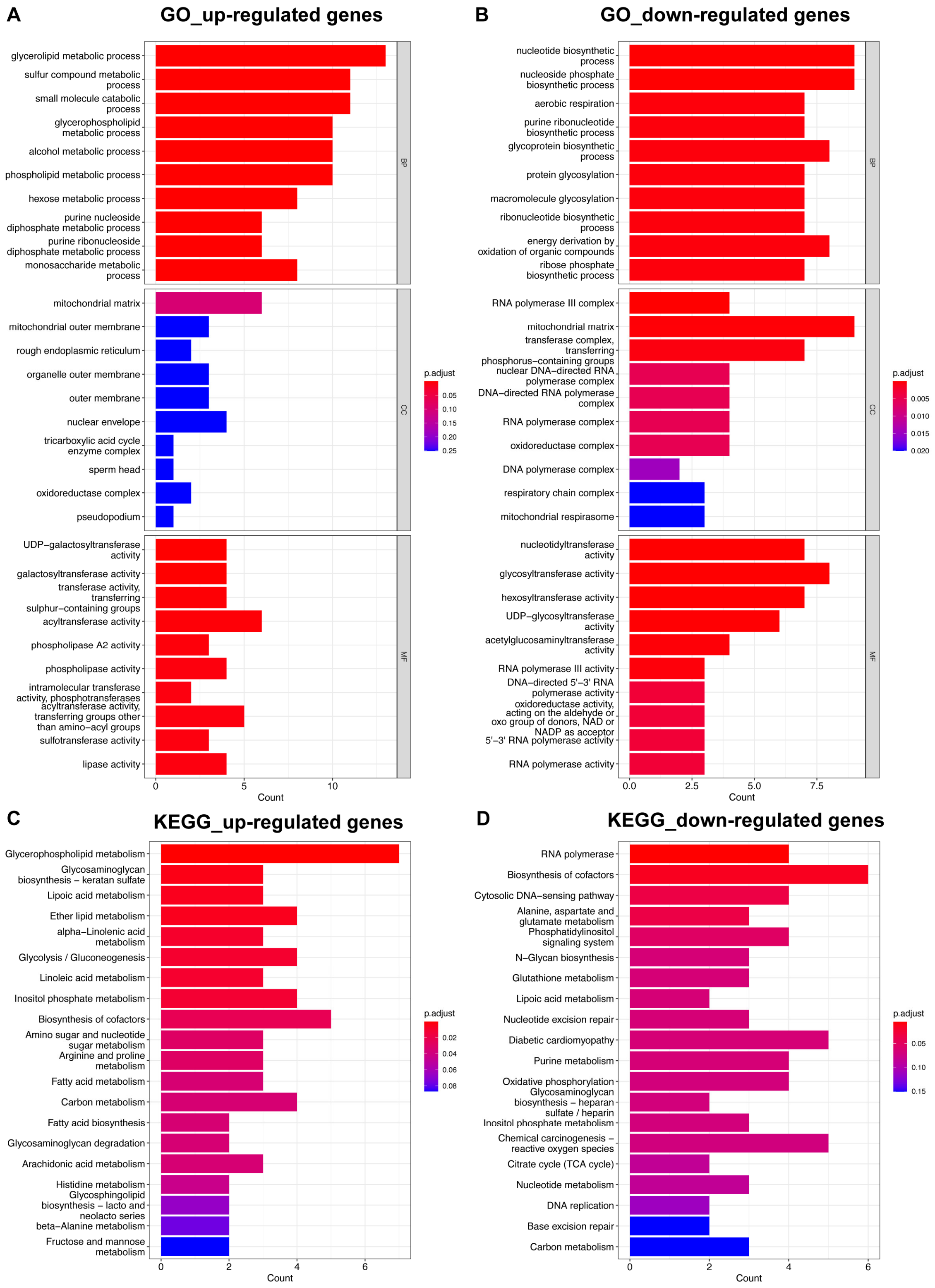

Figure S4. Functional enrichment analyses of separated upregulated and downregulated metabolism-related DEGs. GO enrichment analyses of (A) upregulated and (B) downregulated metabolism-related DEGs. KEGG pathways enriched by (C) upregulated and (D) downregulated metabolism-related DEGs. Each term's P value is colored according to the legend. Abbreviations: BP: biological process, CC: cellular component, DEG: differentially expressed genes, GO: Gene Ontology, MF: molecular function, KEGG: Kyoto Encyclopedia of Genes and Genomes, RIF: recurrent implantation failure.

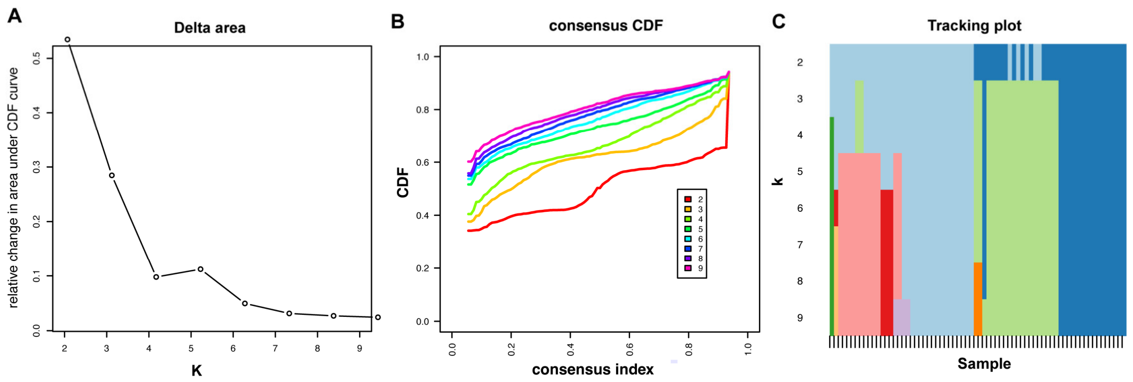

Figure S5. Two metabolic subtypes characterized by different metabolic features and molecular mechanisms. (A) Relative alterations in the area under CDF curve. (B) Consensus CDF when K = 2-9. (C) Tracking plot showing the sample classification when K = 2-9. Abbreviations: CDF: cumulative distribution function.

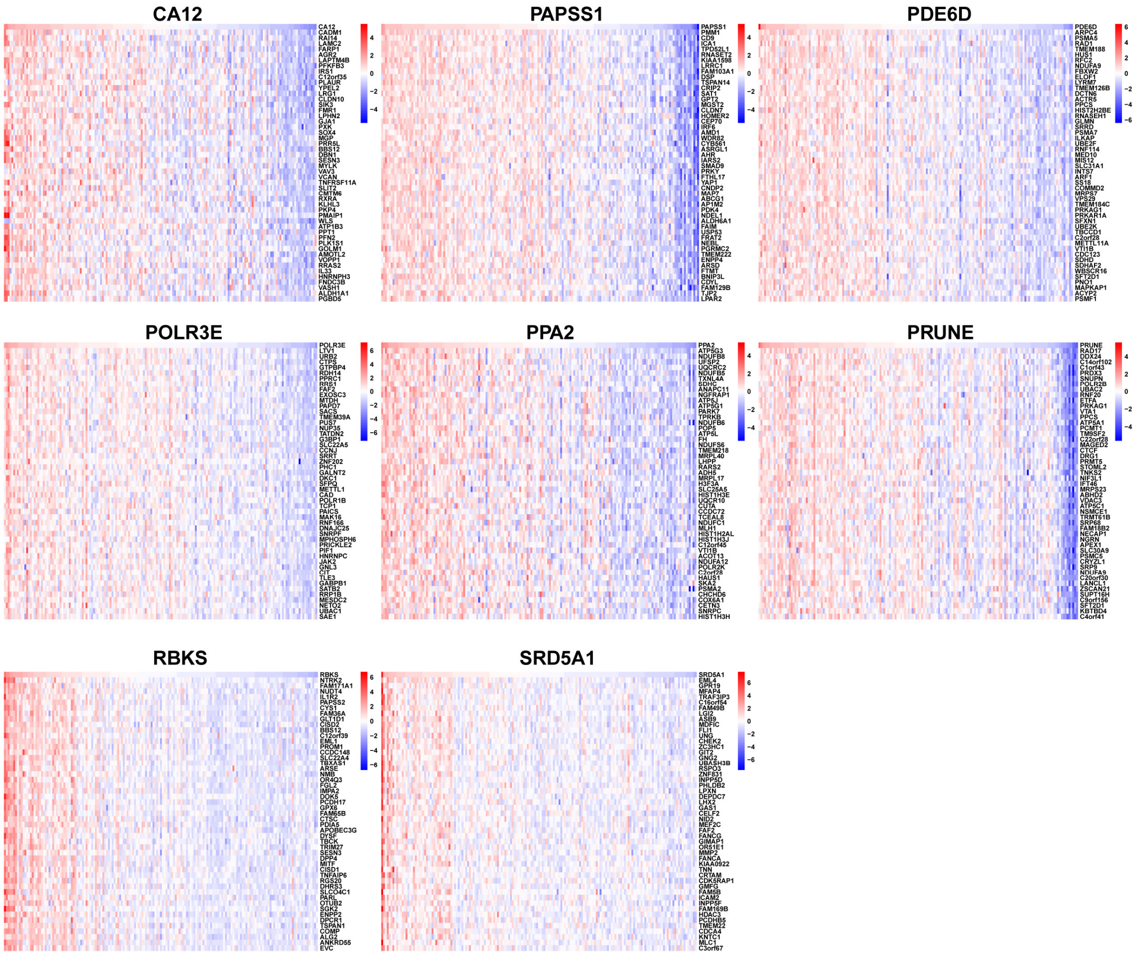

Figure S6. Correlation analysis between eight characteristic genes and all genes from three datasets. Heatmaps of the 50 genes with the strongest correlation.
